# A Theoretical Exploration of Birhythmicity in the p53-Mdm2 Network

**DOI:** 10.1371/journal.pone.0017075

**Published:** 2011-02-14

**Authors:** Wassim Abou-Jaoudé, Madalena Chaves, Jean-Luc Gouzé

**Affiliations:** COMORE Project-team, INRIA Sophia Antipolis, Sophia Antipolis, France; University of Sheffield, United Kingdom

## Abstract

Experimental observations performed in the p53-Mdm2 network, one of the key protein modules involved in the control of proliferation of abnormal cells in mammals, revealed the existence of two frequencies of oscillations of p53 and Mdm2 in irradiated cells depending on the irradiation dose. These observations raised the question of the existence of birhythmicity, i.e. the coexistence of two oscillatory regimes for the same external conditions, in the p53-Mdm2 network which would be at the origin of these two distinct frequencies. A theoretical answer has been recently suggested by Ouattara, Abou-Jaoudé and Kaufman who proposed a 3-dimensional differential model showing birhythmicity to reproduce the two frequencies experimentally observed. The aim of this work is to analyze the mechanisms at the origin of the birhythmic behavior through a theoretical analysis of this differential model. To do so, we reduced this model, in a first step, into a 3-dimensional piecewise linear differential model where the Hill functions have been approximated by step functions, and, in a second step, into a 2-dimensional piecewise linear differential model by setting one autonomous variable as a constant in each domain of the phase space. We find that two features related to the phase space structure of the system are at the origin of the birhythmic behavior: the existence of two embedded cycles in the transition graph of the reduced models; the presence of a bypass in the orbit of the large amplitude oscillatory regime of low frequency. Based on this analysis, an experimental strategy is proposed to test the existence of birhythmicity in the p53-Mdm2 network. From a methodological point of view, this approach greatly facilitates the computational analysis of complex oscillatory behavior and could represent a valuable tool to explore mathematical models of biological rhythms showing sufficiently steep nonlinearities.

## Introduction

Periodic phenomena are encountered at all levels of biological organization, with periods ranging from fractions of a second to years [1]. At the intracellular level, periodic phenomena have been reported in various biochemical systems such as calcium signalling, circadian rythms, cell cycle, glycolysis, cAMP signaling in *Dictyostelium* [1], [2] or the p53-Mdm2 network [3]. Most of the time, biochemical oscillations display a simple pattern with a single oscillatory regime of stable period and amplitude. However, rhythmic processes can sometimes present a more complex behavior. One mode of complex oscillatory behavior is the coexistence of two simultaneously stable oscillatory regimes for the same external conditions. This phenomenon, called birhythmicity [1], [4], is the counterpart of bistability for oscillatory dynamics. Such a behavior has been observed in a number of chemical oscillatory systems [5], [6] but, although some studies suggest its occurrence in the heart and the neuronal system [7], birhythmicity has not yet been firmly observed experimentally in biological systems.

**Figure 1:**
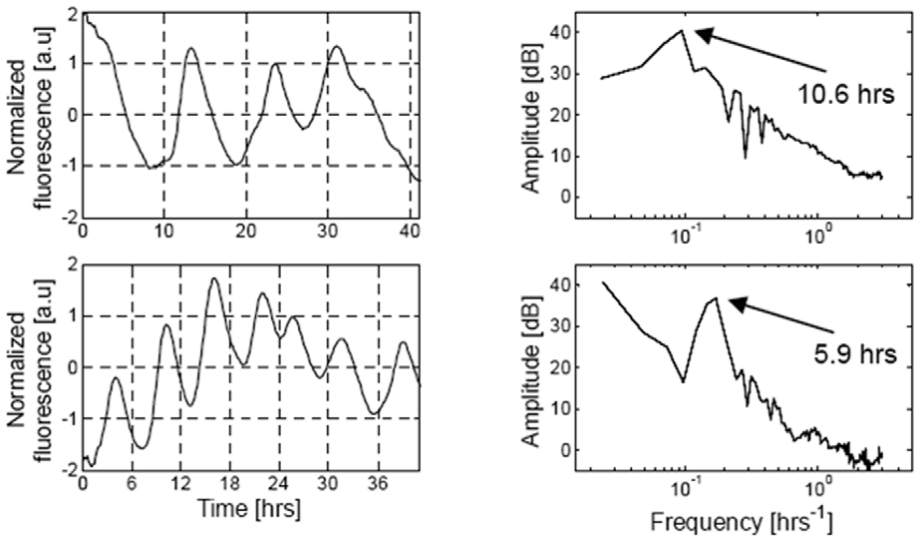
Experimental data from (Geva-Zatorsky et al., 2006, Fig.S3) [11]. Power spectrum of nuclear Mdm2-YFP fluorescence dynamics in individual cells. Top: an example of a cell showing fluctuations with a characteristic frequency of ∼10 hours (exposed to 0.3Gy of gamma irradiation), and the power spectrum of the signal (by Fourier transform). Bottom: an example of a cell with multiple oscillations with a period of ∼6 hours (exposed to 5Gy), and the power spectrum of the signal (right). Reprinted by permission from Macmillan Publishers Ltd: Molecular Systems Biology, advance online publication, 2006 (doi: 10.1038/msb4100068).

Recent experiments performed in the p53-Mdm2 network, one of the key protein module involved in the control of proliferation of abnormal cells in mammals [8], [9], [10], reported two oscillatory regimes of p53 and Mdm2 in irradiated cells [11]: a low-frequency oscillatory regime at low irradiation dose with a period of about 10 h and high-frequency oscillations at high irradiation dose with a period of about 6 h (Figure 1). This observation raised the question of the existence of birhythmicity in the p53-Mdm2 network which would be at the origin of the two oscillatory regimes experimentally observed as a function of the irradiation dose.

A theoretical answer to this question has been recently proposed by Ouattara, Abou-Jaoudé and Kaufman [12], [13]. In the framework of a simple 3-dimensional differential model of the p53-network, they showed that this system could display birhythmicity for a certain range of irradiation dose [12]. The simultaneous presence of two distinct periodic orbits allowed the model to reproduce in particular (i) the two frequencies experimentally observed, (ii) the increase of the fraction of cells oscillating with a high frequency when the irradiation dose increases and (iii) the changes in the oscillation frequency that have been observed for some cells after irradiation [12], [13].

Following this work, the aim of this paper is to investigate the mechanisms at the origin of birhythmicity in Ouattara, Abou-Jaoudé and Kaufman's model (OAK model). As this 3-dimensional continuous non-linear differential model is difficult to analyze, in a first step, we approximated it by a 3-dimensional piecewise linear differential model where the Hill functions have been replaced by step functions and, in a second step, by a 2-dimensional piecewise linear differential model by setting one autonomous variable as a constant in each domain of the phase space delimited by the thresholds of the step functions. Analyzing the dynamics of the system in the framework of a piecewise linear differential description allowed us to reveal the phase space structure of the two oscillatory regimes composing the birhythmic behavior.

Following this analysis, we find that two features related to the phase space structure of the system are at the origin of birhythmicity observed in the OAK model: (1) the existence of two embedded cycles in the transition graph of the piecewise differential models; (2) the presence of a bypass containing two folds in the orbit of the large amplitude oscillatory regime of low frequency. Calculation of a first return map further permitted to give an interpretation of the role of these two features in the emergence of the birhythmic phenomenon. Finally, from this analysis, an experimental strategy is proposed to test the existence of birhythmicity in the p53-Mdm2 network.

## Results

### Birhythmicity in the OAK Model

**Figure 2:**
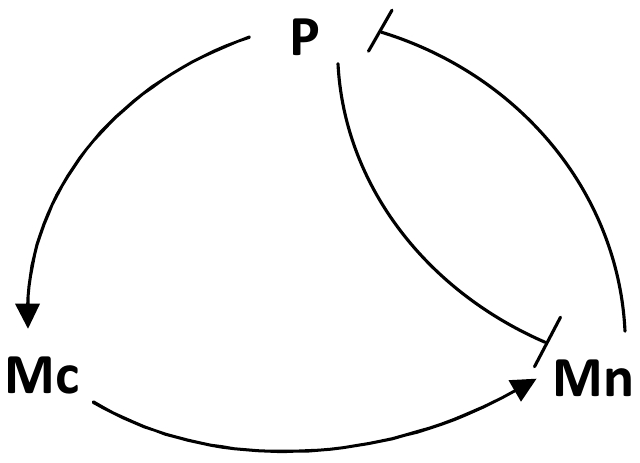
Schematic representation of the p53-Mdm2 core network. Normal arrows correspond to positive interactions, blunt arrows to negative interactions. P, Mc and Mn represent p53, cytoplasmic Mdm2 and nuclear Mdm2 respectively.

The model of the p53-Mdm2 core network proposed by Ouattara, Abou-Jaoudé and Kaufman is a three-variable model, inspired by the work of Ciliberto et al. [14], which describes the interaction between p53, cytoplasmic Mdm2 and nuclear Mdm2. We briefly recall the biological data from which this model has been elaborated (see [12], for a more detailed description). Nuclear Mdm2 down regulates p53 by accelerating its degradation through ubiquitination [15]–[17] and by blocking its functional activity [18]–[20]. p53 up regulates cytoplasmic Mdm2 level by enhancing the transcription of gene *MDM2* [21], [22]. p53 inhibits the translocation of Mdm2 from the cytoplasm to the nucleus [23]. These interactions are summarized by the influence diagram shown in Figure 2. Importantly, the degradation rate of nuclear Mdm2 (d_Mn_) depends on DNA damage caused upon cell irradiation [24], [25] and increases when DNA damage increases [13].

To describe the dynamics of this network, Ouattara, Abou-Jaoudé and Kaufman built a three-dimensional differential model (which will be called the OAK Model from now on) to represent the temporal evolution of the level of p53, cytoplasmic and nuclear Mdm2. We recall the equations of the OAK Model in Text S1. For appropriate parameter values, a bifurcation analysis of this model as a function of d_Mn_ showed the existence of a domain of birhythmicity separating two oscillatory regimes of different amplitude, period and mean values (see Figure 2b in [13], and Figure S1) which allowed to reproduce the two characteristic periods of p53 oscillations observed experimentally by Geva-Zatorsky et al. as a function of cell irradiation dose (Figure 1).

### A three-dimensional reduced model

Starting from the OAK model, we first performed an analysis of the different terms involved in the equations of this model to characterize their contribution to the oscillatory dynamics of the system, in particular to the birhythmic behavior. Our analysis suggests that the terms modeling the translocation process of Mdm2 from the nucleus to the cytoplasm and the bilinear term modeling Mdm2-mediated acceleration of p53 degradation would have little influence on the oscillatory dynamics (see Text S2). Suppressing these terms, the OAK Model becomes:
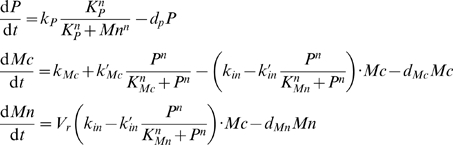
(Model 1)where P, Mc and Mn represent the concentration of p53, cytoplasmic Mdm2 and nuclear Mdm2 respectively.

**Figure 3:**
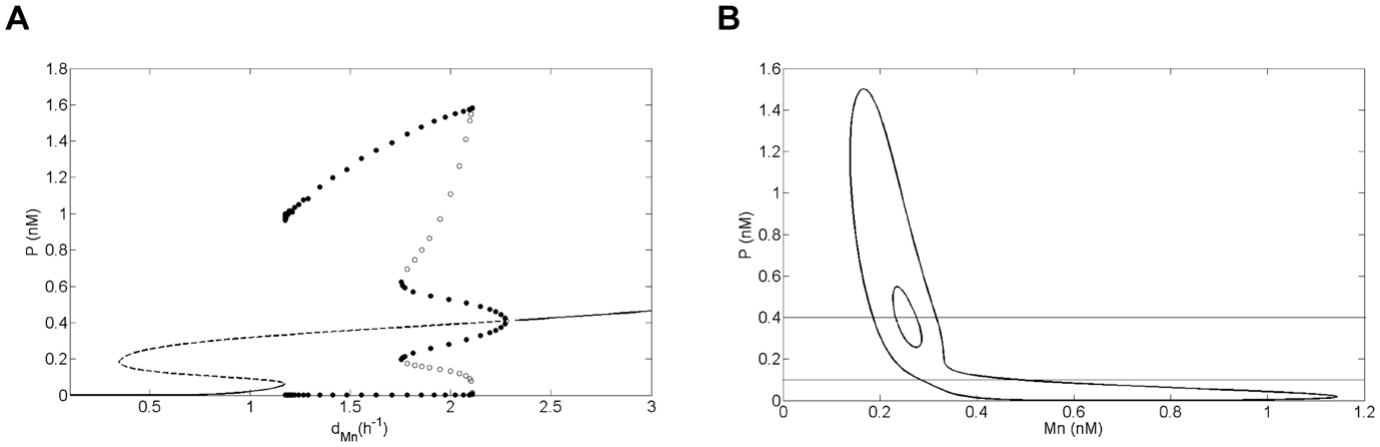
Bifurcation diagram and projection of the phase portrait of birhythmicity in the plane (Mn,P) for Model 1. (A) Bifurcation diagram of p53 level as a function of d_Mn_ for Model 1. Solid lines (resp. dashed lines) represent the stable (resp. unstable) equilibrium points. Bold (resp. white) dots are the maxima and minima of the stable (resp. unstable) limit cycles. The system shows a birhythmic domain for 1.75 h^−1^<d_Mn_<2.11 h^−1^. (B) Projection of the two oscillatory regimes on the plane (Mn,P) for Model 1 for d_Mn_ = 1.9 h^−1^. The thresholds K_Mc_ and K_Mn_ related to P are indicated in solid lines. The parameter values of Model 1 are the same as for the OAK Model (K_Mn_ = 0.1 nM) except: n = 6, d_P_ = 2.5 h^−1^ and K_Mc_ = 0.4 nM (see legend of Figure S1 for the parameter values of the OAK Model).

Numerical simulations of Model 1 show that, for appropriate parameter settings, the bifurcation diagram as a function of d_Mn_ is very similar to the bifurcation picture of the OAK Model if the non-linear terms are slightly steeper (i.e. higher Hill coefficients n) (Figure 3A, and Figure S1). As d_Mn_ increases, Model 1 displays successively a stable steady state with a low level of p53, the coexistence of this stable steady state with two unstable steady states, a stable oscillatory state, and finally a stable steady state with high levels of p53. In the oscillatory domain, Model 1 shows two limit cycles, a large amplitude oscillatory regime of low frequency for low d_Mn_ values and a small amplitude oscillatory regime of high frequency for high d_Mn_ values, separated by a birhythmic region where these two regimes coexist (Figure 3A). In the domain of birhythmicity, numerical simulations moreover show that the phase portrait of the two limit cycles for the OAK Model is very similar to the one for Model 1 (Figure 3B, and Figure S1). In the remainder of this work, Model 1 will thus be used as the starting model to investigate the mechanisms leading to the emergence of the birhythmic behavior in the OAK Model.

We next qualitatively analyzed the main differences between the two oscillatory regimes for Model 1. Our analysis shows significant differences between the shape of the orbits of the two oscillatory regimes in the phase space (Figure 3B). Indeed:

- the small amplitude oscillatory regime of short period has a regular shape. Moreover, p53 level stands well above K_Mn_ threshold all along the trajectory of the cycle. The Hill function 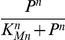 modeling the inhibition of Mdm2 translocation into the nucleus by p53 has thus no significant impact along this cycle 
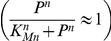
.
- on the contrary, the large amplitude oscillatory regime of long period contains a long “tail” in the phase space domain where p53 is below K_Mn_ threshold and its orbit crosses the two thresholds, K_Mn_ and K_Mc_, related to P.

Finally, we performed an analytical study of the dynamics of the system by investigating general properties concerning the existence of limit cycles. Contrary to 2-dimensional differential systems where the Poincaré-Bendixson theorem gives general conditions for the existence of periodic orbits, there is no such equivalent theorem concerning the existence of periodic orbits for 3-dimensional differential system except for particular systems such as competitive or cooperative systems [26]. In the case of Model 1, an analysis of the sign of the elements of the Jacobian Matrix interestingly shows that this model is indeed equivalent to a competitive system (Text S3). According to Hirsch [27], a Poincaré-Bendixson theorem in dimension 3 holds. Moreover, for the parameter values for which the system displays birhythmicity, a numerical study of the equilibrium points suggests that there is only one equilibrium point which is unstable and for which the stable manifold is 1-dimensional (Text S3 and Figure S2). Applying the Poincaré-Bendixson theorem for 3-dimensional competitive systems, it can be proved that the system admits a periodic orbit for the parameter values for which the system displays birhythmicity [26]. Under some supplementary assumptions satisfied by our system, we can moreover show that there exists a stable periodic orbit [28]. However, these theorems do not tell how many periodic orbits the system has and thus do not provide a mathematical proof of the existence of birhythmicity in our model.

### From continuous to discrete model: approximation of the Hill functions by step functions

Although we can derive some general properties concerning the dynamics of the 3-dimensional differential Model 1, it is still difficult to get insight into the dynamics of the system, in particular into the birhythmic behavior.

However, the equations of Model 1 involve several highly non-linear terms of the Hill form:


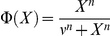
 for increasing Hill functions;


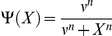
 for decreasing Hill functions.

**Figure 4:**
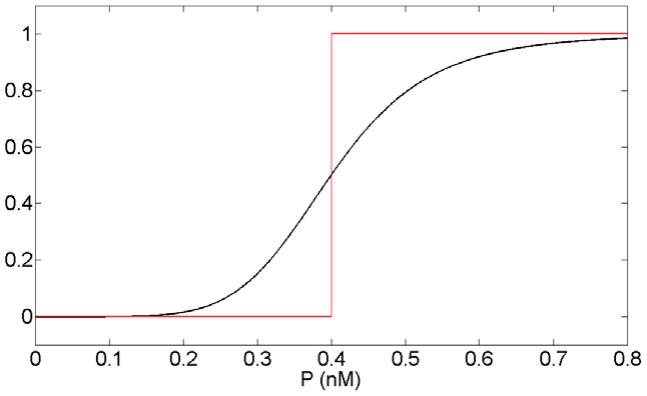
Approximation of Hill function to step function. Hill function 
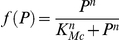
which appears in the equation of Mc in Model 1 (in black) and its approximation into the step function 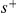 defined as: 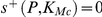 if 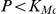 and 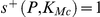 if 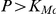 (in red) for n = 6 and K_Mc_ = 0.4 nM. n is the Hill coefficient and characterizes the steepness of the Hill function.

We thus approximated Model 1 by replacing these terms by step functions with two levels (Figure 4):
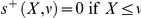


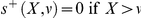
for increasing Hill functions and:
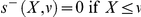


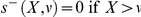
for decreasing Hill functions, where ν represents the threshold of the step functions.

Such an approximation has been originally proposed by Glass and Kaufman [29] for biochemical networks and has been since widely used to model genetic systems [30]–[33]. This approximation leads to a piecewise linear differential model whose equations are:
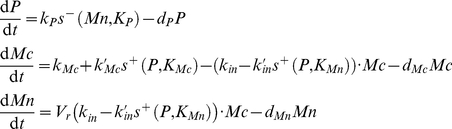
(Model 2)


**Figure 5:**
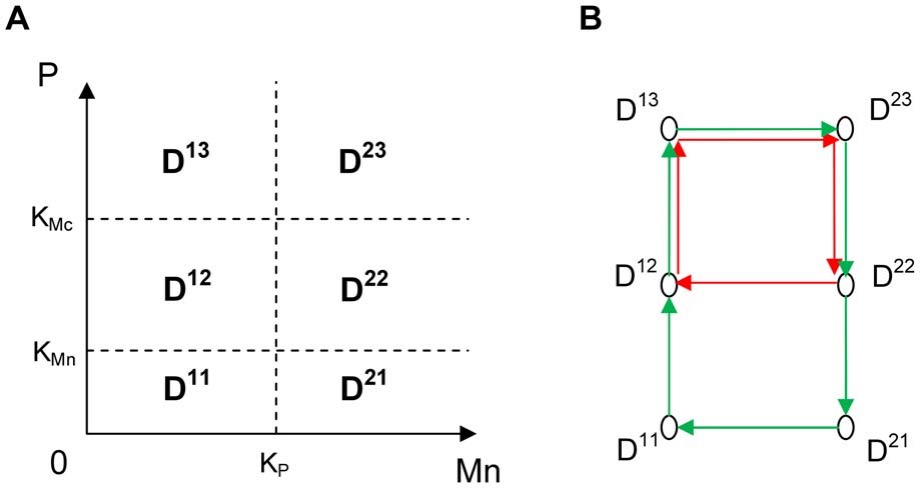
Subdivision of the phase space and graph of transitions for Model 2. (A) Subdivision of the phase space for Model 2 in 6 domains delimited by the thresholds K_P_, K_Mn_ and K_Mc_. (B) Graph of the transitions followed by the two oscillatory regimes composing birhythmicity shown in Figure 6. The small amplitude oscillatory regime of short period crosses domains D^22^, D^12^, D^13^ and D^23^ successively (in red). The large amplitude oscillatory regime of long period crosses domains: D^22^, D^21^, D^11^, D^12^, D^13^ and D^23^ successively (in green).

The space of variables can thus be decomposed into 6 domains (D^11^, D^21^, D^12^, D^22^, D^13^, D^23^) delimited by the threshold values of the step functions: K_P_, K_Mn_ and K_Mc_ (Figure 5A). The equations of evolution in each domain of the phase space are detailed in Table S1. In each domain, the equations are affine and stable and one can calculate a so-called *target equilibrium point* of the domain towards which the system will tend (Table S2). The *target equilibrium point* is an analogous of the *focal point* defined in a class of piecewise linear diagonal models [34] (see next section). If a target equilibrium point of a domain D^ij^ does not belong to its domain, then the system starting from D^ij^ will leave D^ij^ at some time as it will reach sooner or later a boundary of the domain. If a target equilibrium point of a domain D^ij^ belongs to D^ij^, it corresponds to a stable equilibrium point of the system. Importantly, the equation of Mn depends on Mc. It follows that the sign of the derivative of Mn can change in each domain of the phase space according to Mc. The *transition graph*, which is the graph defining the possible transitions between the different domains [35], can thus not be directly derived from the position of the target equilibrium points (see next section).

**Figure 6:**
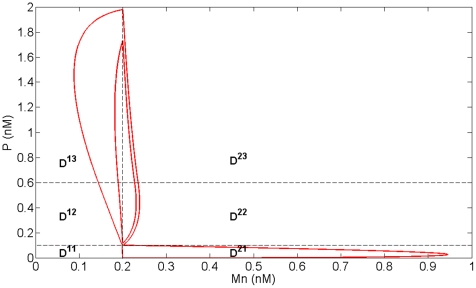
Projection of the phase portrait of birhythmicity in the plane (Mn,P) for Model 2. Projection of the two oscillatory regimes composing the birhythmic behavior of Model 2 in the plane (Mn,P). The dashed lines represent the thresholds of the step functions: K_Mn_ and K_Mc_ for P, K_P_ for Mn. The parameter values are the same as for Figure 3 (K_Mn_ = 0.1 nM, K_P_ = 0.2 nM) except K_Mc_ = 0.6 nM. The period of the large amplitude oscillatory regime is significantly longer than the period of the small amplitude oscillatory regime (see Figure 8A and 8C).

For appropriate parameter settings, numerical simulations show that the bifurcation diagram as a function of d_Mn_ is similar to the bifurcation picture of Model 1: a stable steady state of low p53 values for low d_Mn_ values corresponding to the target equilibrium point of domain D^21^ (P = 0, 
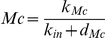
, 
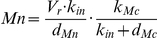
), a stable steady state of high p53 levels for high d_Mn_ values corresponding to the target equilibrium point of domain D^13^ (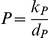, 
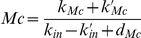
, 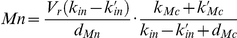) and an oscillatory regime for intermediate d_Mn_ values (see Text S4 and Figure 6). In the oscillatory region, there exists a range of d_Mn_ values for which the system displays birhythmicity with the coexistence of two oscillatory regimes of different amplitude and period (Figure 6):

- a small amplitude oscillatory regime of short period, crossing domains D^12^, D^22^, D^13^ and D^23^, in which the K_Mn_ threshold is not functional (i.e. P>K_Mn_ along the orbit). This periodic orbit corresponds to the small amplitude oscillatory regime appearing in Model 1 (Figure 3B);
- a large amplitude oscillatory regime of long period passing through all the domains of the phase space. This periodic orbit corresponds to the large amplitude oscillatory regime of Model 1 (Figure 3B) and contains a broad outward excursion in domain D^21^, in the form of a “tail”. As also observed for Model 1, this “tail” contains two folds where the sign of dMn/dt changes: a fold in the transition between D^22^ and D^21^ and a fold inside domain D^21^.

The sequence of transitions followed by the small and the large amplitude limit cycles are shown in Figure 5B. As emphasized in the previous section, the orbits of the two limit cycles are significantly different. Using Model 2, we can clearly separate the trajectories of the two limit cycles by decomposing them into a sequence of transitions in the state space. Each of the two orbits evolves in a dynamical regime with distinct qualitative properties and distinct biological consequences. Starting from the threshold delimiting domain D^12^ and D^13^ (i.e. p53 levels in its highest threshold K_Mc_ and nuclear Mdm2 levels is below its threshold K_P_), the two oscillatory regimes can be described as follows. As p53 level goes above the K_Mc_ threshold, p53 starts to activate the expression of gene *MDM2* and thus the production of cytoplasmic Mdm2. Mdm2 is then translocated into the nucleus where it accumulates. When nuclear Mdm2 level gets beyond its threshold K_P_, it starts to inhibit p53 activity (domain D^23^). p53 level then decreases. As its level goes below its threshold K_Mc_, p53 no longer activates the expression of *MDM2*. Cytoplasmic Mdm2 thus starts to decrease leading to a decrease of nuclear Mdm2 level (domain D^22^). As p53 and nuclear Mdm2 levels both decrease, two alternatives can be chosen by the system leading to either the small or the large amplitude oscillatory regime, which corresponds to the branching point in the graph shown in Figure 5B:

- either nuclear Mdm2 level first goes below its threshold K_P_. Nuclear Mdm2 then no longer inhibits p53 and p53 level starts to increase again (domain D^12^) until it reaches its threshold K_Mc_ which closes the trajectory of the small amplitude limit cycle.
- or p53 level first goes below its threshold K_Mn_. p53 then no longer inhibits Mdm2 entry into the nucleus (domain D^21^) and nuclear Mdm2 starts to rise again, leading to the first fold observed in the transition between D^22^ and D^21^ in the large amplitude limit cycle. However, at the same time, cytoplasmic Mdm2 decreases because p53 level is too low to activate *MDM2* transcription. When cytoplasmic Mdm2 level gets too low to feed nuclear Mdm2, nuclear Mdm2 level then starts to decrease leading to the second fold observed in the large amplitude limit cycle in domain D^21^. As nuclear Mdm2 level goes below its threshold K_P_, nuclear Mdm2 no longer inhibits p53 activity whose level starts to increase again (domain D^11^ and D^12^) until it reaches its threshold K_Mc_ which closes the trajectory of the large amplitude limit cycle.

The analysis of the orbits of the two oscillatory regimes thus reveals two key qualitative features which characterize the birhythmic behavior:

- the choice between two transitions when the system reaches domain D^22^ which induces a separation in the phase space between the two oscillatory regimes. This branching point gives rise to two embedded cycles in the graph of transitions representing the two oscillatory regimes in the phase space (Figure 5B). This result is in accordance with the logical analysis of the p53-Mdm2 network which brought out an oscillatory regime composed of several small and large amplitude embedded cycles in the transition graph of the system (see Figure 3 in [12]);
- the presence of a “tail” containing two folds in the orbit of the high amplitude oscillatory regime (Figure 6, domain D^21^) whose emergence can be interpreted in terms of the competition between two opposite processes: Mdm2 translocation from the cytoplasm to the nucleus on one hand and p53-mediated inhibition of Mdm2 nuclear entry on the other hand.

### Analysis of the basic mechanisms for the emergence of birhythmicity

#### A two-dimensional reduced model

In order to get more insight into the basic mechanisms leading to birhythmicity, we looked for a further approximation of Model 2 which would preserve the birhythmic behavior as well as the characteristics of the orbits of the two oscillatory regimes underlined by the previous analysis. In Model 2, in each domain, the evolution of Mn depends on Mc whereas the evolution of Mc does not depend on the other variables of the system (Table S1). We thus considered Mc as a forcing external parameter applied on the evolution of Mn. In this respect, we set Mc as a constant Mc^ij^ in each domain D^ij^ of the phase space under some constraints on the Mc^ij^ values which will be detailed further.

This approximation leads to a 2-dimensional piecewise linear differential model with the phase space delimited by the thresholds K_Mc_, K_Mn_ and K_P_. The evolution of each variable in each domain is of the form:
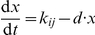
where x represents the level of P or Mn, k_ij_ is a constant which depends on the domain D^ij^ of the phase space and d is the degradation rate of P or Mn. This type of piecewise diagonal linear systems belongs to a class of dynamical systems proposed originally by Glass and Kaufman [29]. Interestingly, these systems have mathematical properties which favor the qualitative analysis of their dynamics [35]–[38]. In particular, one can derive the so-called *transition graph*, which describes all the possible transitions between the different domains, from the position of the target equilibrium points of each domain (called the *focal points*) [31], [35], [36], [38].

**Figure 7:**
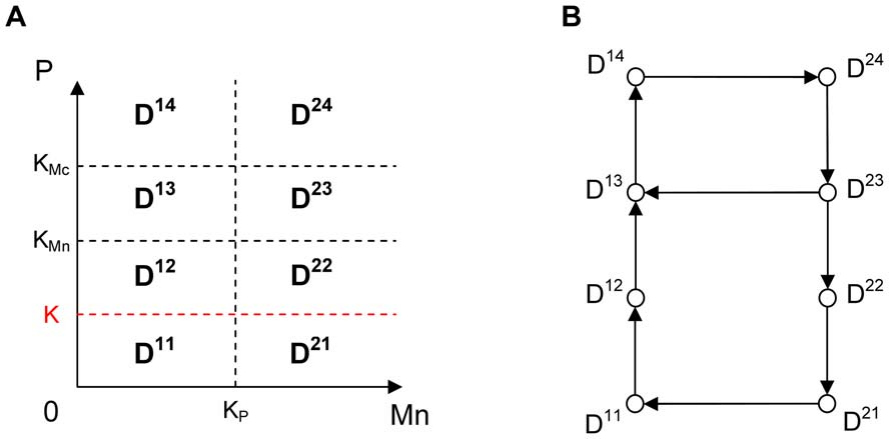
Subdivision of the phase space and transition graph for Model 3. (A) Subdivision of the phase space for Model 3 in 8 domains delimited by the thresholds K_P_, K_Mn_, K_Mc_ and the additional threshold K (in red). (B) Transition graph of Model 3. The graph contains a branching point in domain D^23^ and two embedded cycles. From this domain, the system can either go to domain D^13^ or domain D^22^ depending on the initial conditions.

In order to reproduce the two folds forming the outward excursion which characterizes the large amplitude oscillatory regime in Model 1 and Model 2, we added another threshold K (K<K_Mn_) for p53 level. As the evolution of Mn in each domain is now monotone, the introduction of this new threshold allows changing the sign of the derivative of Mn as the system crosses threshold K and thus recovering in particular the fold observed in domain D^21^ in Model 2 (Figure 6). The space of variables can thus be decomposed into 8 domains (D^11^, D^21^, D^12^, D^22^, D^13^, D^23^, D^14^, D^24^), defined by the thresholds K_Mc_, K_Mn_ plus the additional threshold K for p53, and K_P_ for nuclear Mdm2 (Figure 7). The equations of evolution in each domain of the phase space are detailed in Table S3 (Model 3).

**Figure 8:**
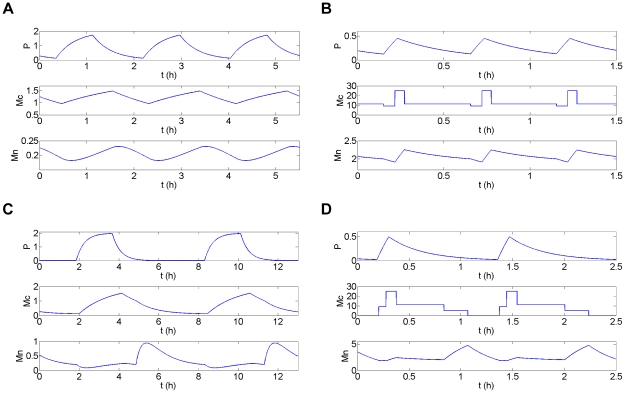
Temporal simulations for Model 2 and Model 3. Temporal simulation (h) of the concentration (in nM) of p53 (P), cytoplasmic Mdm2 (Mc) and nuclear Mdm2 (Mn) for Model 2 (A and C) and Model 3 (B and D) in the small amplitude short period (A and B) and the large amplitude long period (C and D) oscillatory regime. For Model 3, Mc has been set as a constant Mc^ij^ in each domain D^ij^ of the phase space following constraint (2) (see text). The parameter values are indicated in Figure 6 for Model 2 and in Figure 9 for Model 3.

Moreover, in order to keep the basic characteristics of the birhythmic behavior shown in Model 2, we imposed the following constraints on the parameters values of Model 3 (see Table S4 and Text S5 for more details):

- constraint (1): the transition graph of Model 3 contains the sequence of transitions corresponding to the two oscillatory regimes in Model 2 (Figures 5 and 7).
- constraint (2): the setting of the parameters Mc^ij^ in each domain D^ij^ of the phase space of Model 3 has to be in accordance with the evolution of Mc in the two oscillatory regimes from one domain to another in Model 2 (Figure 8).

Finally, in order to simplify the analytical calculation of the return map (see section on the analysis of the first return map), the values of the degradation rate, d_Mn_ and d_P_, have been chosen to be the same. In this case, the trajectories in each domain of the phase space are straight lines, which greatly reduce the explicit computation of the return map. However, considering different degradation rate values does not change the main results of our analysis. Indeed, we can still numerically recover birhythmicity and the basic characteritics of this behavior for nonequal degradation rates (not shown).

#### Numerical simulations of the 2-dimensional model

**Figure 9:**
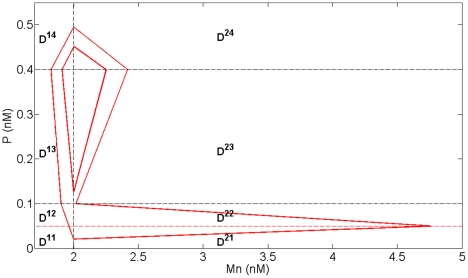
Phase portrait of birhythmicity for Model 3. Simulation of the two oscillatory regimes in the phase space for Model 3. The dashed lines represent the thresholds K_Mc_, K_Mn_ and K for P, K_P_ for Mn. The parameter values are the same as for Figure 6 (K_Mn_ = 0.1 nM) except K_Mc_ = 0.4 nM, K = 0.05 nM, K_P_ = 2 nM, d_P_ = 3 h^−1^, Mc^11^ = Mc^21^ = Mc^12^ = 0, Mc^22^ = 5 nM, Mc^13^ = 9 nM, Mc^23^ = 11.3 nM, Mc^14^ =  Mc^24^ = 25 nM. Note that, since the degradation constants d_Mn_ and d_P_ have the same values, the trajectories in each domain are straight lines.

**Figure 10:**
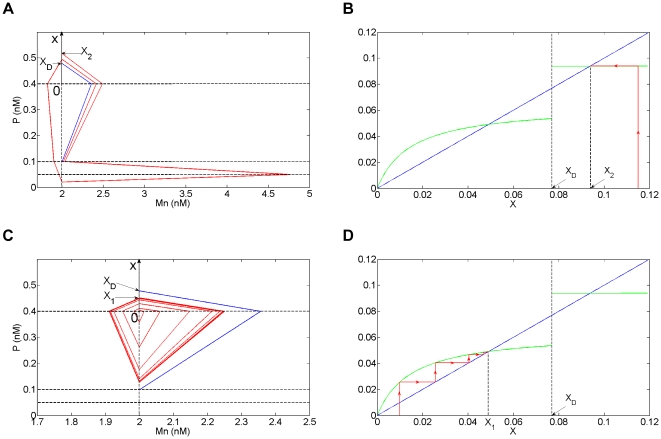
First return map analysis for Model 3. (A and C) In red, simulation in the phase plane for the initial conditions Mn = 2 nM and P = 0.515 nM (x>x_D_, panel A) or P = 0.401 nM (x<x_D_, panel C) for Model 3. The dashed lines represent the thresholds K_Mc_ = 0.4 nM, K_Mn_ = 0.1 nM and K = 0.05 nM for P, K_P_ = 2 nM for Mn. (B and D) In red, simulation of the orbit of the first return map from and to the [0, x) axis for the initials conditions of panel A (panel B) and panel C (panel D). The first return map F is shown in green and has been derived analytically (see Text S6). The parameter values are indicated in Figure 9. The discontinuity in F at x = x_D_∼0.077 arises when the trajectory hits the threshold intersection point (Mn = K_P_ = 2, P = K_Mn_ = 0.1) (blue curve). The fixed points of F correspond to the two limit cycles of the system shown in Figure 9. For x>x_D_ (resp. x<x_D_), the trajectory tends to the large (resp. small) amplitude oscillatory regime corresponding to the fixed point x = x_2_∼0.093 (resp. x = x_1_∼0.048).

For appropriate parameter settings respecting the constraints stated above, numerical simulations show that the system exhibits a birhythmic behavior whose phase portrait is similar to the projection in the plane (Mn,P) of the phase portrait of the birhythmic behavior observed in Model 2 (Figure 9). In accordance with constraint (1), the small amplitude limit cycle crosses domains D^14^, D^24^, D^23^ and D^13^ whereas the large amplitude limit cycle passes through all the domains of the phase space. As shown in the transition graph (Figure 7B), when the system reaches domain D^23^, it can either go to D^13^ or D^22^. This branching point thus induces a separatrix in D^23^, passing through the threshold intersection (Mn = K_P_, P = K_Mn_) and the focal point of D^23^, which delimits the basins of attraction of the two oscillatory regimes of the birhythmic behavior (Figure 10A and 10C, blue curve). Finally, the large amplitude cycle contains a “tail” with two folds which appear at two domain transitions: D^23^ to D^22^ when the level of P crosses K_Mn_ and D^22^ to D^21^ when the level of P level crosses the additional threshold K. These folds reproduce the two folds observed in the large amplitude limit cycle in Model 2 (see Figure 6).

#### Analysis of a first return map of the 2-dimensional model

We next looked for a proof of the existence of birhythmicity by analyzing the first return map from and to the boundary between the domains D^14^ and D^24^ ([0, x) axis in Figure 10) which is crossed by both oscillatory regimes. The return map (or Poincaré map) of a surface S (here the [0, x) axis) is a mapping of S obtained by following the trajectory between two consecutive intersections with S [39]. This mapping is described by the equation:
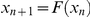
where x_n_ (resp. x_n+1_) represents the n^th^ (resp n+1^th^) intersection of the trajectory of the system with S, and F represents the first return map. In this description, the periodic orbits crossing S correspond to fixed points of the function F (i.e. points x for which 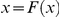).

Here, an analytical expression of the first return map of the [0, x) axis can be calculated (see Text S6). The results of our analysis show that the return map (Figure 10B and 10D, green curve) presents two strictly positive fixed points which correspond to the two oscillatory regimes shown by the numerical simulations in Figure 9, proving the existence of birhythmicity in Model 3. Moreover and surprisingly, a discontinuity point appears which separates the [0, x) axis in two intervals: the interval [0, x_D_] for which the system tends to the small amplitude oscillatory regime (x = x_1_) (Figure 10C and 10D); the interval x>x_D_ for which the system tends to the large amplitude oscillatory regime (x = x_2_) (Figure 10A and 10B). For the critical point x = x_D_, the trajectory hits the threshold intersection point (Mn = K_P_, P = K_Mn_) (Figure 10A and 10C, blue curve), drawing the separatrix of the system in domain D^23^. Therefore, the branching point of the transition graph, which induces this separatrix, is at the origin of the discontinuity point observed in the return map.

**Figure 11:**
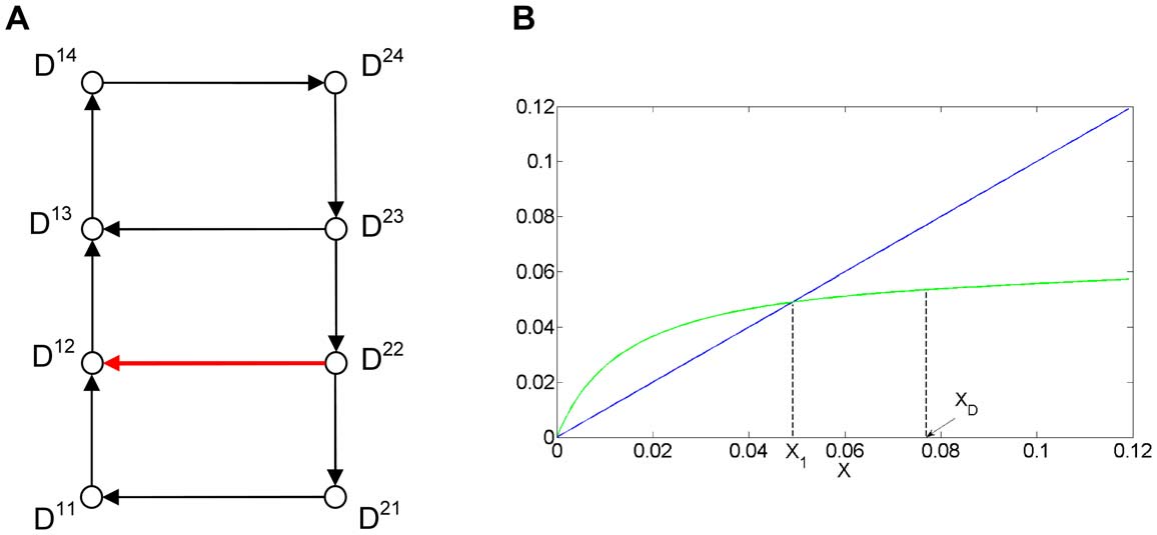
Transition graph and first return map for the modified Model 3. (A) Transition graph of the modified model 3. (B) Graph of the corresponding first return map (in green) from and to the [0, x) axis (see Figure 10). The parameter values are indicated in Figure 9, except Mc^22^ = 1. The focal point of D^22^ is now in D^11^ inducing the additional transition from D^22^ to D^12^ (arrow in red). The fixed points of the first return function correspond to limit cycles of the system. The system presents a unique limit cycle (x = x_1_∼0.048) corresponding to the small amplitude oscillatory regime (see Figure 10C and 10D). The first return map shows a non smooth point at x_D_∼0.077 (see Text S6).

We then investigated the role of the outward excursion which characterizes the large amplitude oscillatory regime in the emergence of the birhythmicity by using the return map description. To do so, we suppressed this “tail”, while keeping the two embedded cycles in the transition graph, by adding a transition from domain D^22^ to domain D^12^ in the transition graph (Figure 11A). We then calculated the first return map in the [0, x) axis for this modified Model 3 (see Text S6 for details of the calculation). The results show that the large amplitude limit cycle disappeared (Figure 11B). Indeed, the first return map ceases to be discontinuous and instead presents a non smooth point for the same x_D_ value than before (Text S6). The loss of the discontinuity point accompanies the loss of the fixed point, x = x_2_, corresponding to the large amplitude oscillatory regime. Therefore, the “tail” is here necessary for the emergence of the birhythmic behavior. More precisely, the suppression of the “tail” leads to the disappearance of the discontinuity of the first return map which has for consequence the disappearance of the large amplitude limit cycle.

### An experimental strategy to reveal birhythmicity in the p53-Mdm2 network

From the previous analysis, an experimental strategy can be proposed to test the existence of the birhythmic behavior predicted by the OAK Model. The experiments performed on irradiated cells by Geva-Zatorsky et al. showed the existence of two frequencies of p53 oscillations as a function of the irradiation dose but the co-existence between these two types of oscillations for the same irradiation dose has not been firmly observed experimentally so far [11]. The 2-dimensional piecewise linear reduced system (Model 3) provides a model of the p53-Mdm2 network from which experimental strategies can be implemented to distinguish between existence of a single oscillatory regime (mono-rhythmicity) whose frequency increases with the irradiation dose, or coexistence of two oscillatory regimes for the same irradiation dose (birhythmicity).

**Figure 12:**
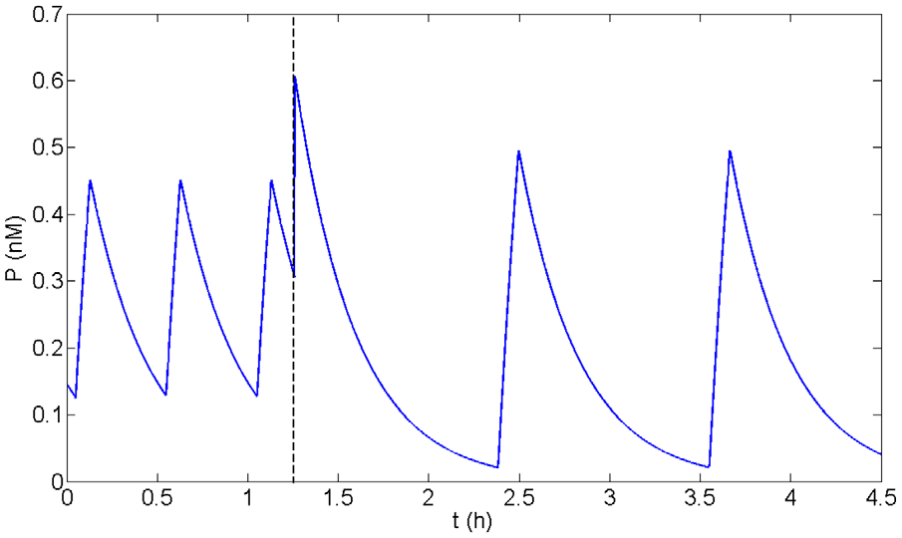
Permanent shift from short to long period oscillatory regime after a transient p53 pulse. Temporal simulation of p53 level for Model 3. A pulse of p53 is applied at t = 1.2 h (dashed line). Before applying the pulse, the system is oscillating in the small amplitude oscillatory regime. The p53 pulse induces a shift from the small amplitude short period to the large amplitude long period limit cycle. The parameter values are indicated in Figure 9.

Looking at the phase portrait of the oscillatory regimes for Model 3 (Figure 9), it's straightforward to predict that, starting from the small amplitude limit cycle of short period, an increase in p53 concentration will lead to a shift of the system to the other oscillatory regime. Indeed, numerical simulations of this model show that applying a transitory pulse of p53 when the system oscillates in the short period oscillatory regime leads to a shift to long period oscillations (Figure 12). It has to be noted that these type of strategies in which a transient pulse of an inducer is applied to provoke a permanent switch from one regime to another have already been implemented to reveal bistable behavior in genetic networks [40]–[42]. We thus propose the following experimental test: introduce an inducible P53 gene into the cells. Then apply to the cells a set of increasing irradiation doses for which most of the cells are oscillating with a high frequency. For each dose, apply a transitory pulse of the inducer (which will induce a transitory expression of the inducible P53 gene) and wait until the distribution of the periods among the cell population reaches a stationary regime. Has the distribution shifted to long period p53 and Mdm2 oscillations? If yes, this experimental observation is consistent with the existence of a birhythmic behavior in the system. However, in order to show the coexistence of two distinct period distributions for the same d_Mn_ values, the DNA repair process triggered by cell irradiation has to be slow (i.e. d_Mn_ decreases slowly as DNA damage is repaired) compared to the time required to reach the stationary regime after a p53 pulse induction. If this assumption is not fulfilled, a more elaborated experiment, in which the repair process would be slowed down, has to be designed.

## Discussion

The aim of this work has been to analyze the mechanisms at the origin of the birhythmic behavior observed in Ouattara, Abou-Jaoudé and Kaufman's differential model (OAK Model) of the p53-Mdm2 network. To do so, the Hill functions have been firstly approximated into step functions leading to a 3-dimensional piecewise linear differential model in which the birhythmic behavior is conserved. An autonomous variable of the system has then been set as a constant in each domain defined by the thresholds of the step functions to obtain a 2-dimensional piecewise linear differential model also displaying birhythmicity.

Converting the sharp non-linearities of the model into discontinuous step functions revealed the phase space structure of the birhythmic behavior. This approximation coupled with a reduction of the dimensionality of the system, from a 3-dimensional to a 2-dimensional system, and then from 2-dimensional to a 1-dimensional first return map description, yielded a reduced model from which the basic qualitative features observed in the phase space leading to birhythmicity have been extracted: (1) the presence of two embedded cycles in the transition graph of the piecewise linear models, (2) the presence of a “tail” in the orbit of the large amplitude oscillatory regime of long period.

By calculating the first return map, we have theoretically shown the existence of birhythmicity in the framework of the 2-dimensional piecewise linear model. This map also elucidates the role of these two features in the emergence of the birhythmic phenomenon: (1) is associated with a non smooth point of the return map whereas (2) leads to a discontinuity in the return map, which is at the origin of the emergence of the birhythmic behavior. In general, on a methodological level, our analysis shows that the mechanisms leading to complex dynamical behavior such as birhythmicity can be studied by developing qualitative models of the system. One major advantage of this approach is to greatly facilitate the computational analysis of the model. In this regard, this method could be implemented to analyze the dynamics of other mathematical models of biological rhythms showing sufficiently steep nonlinearities.

Finally, in the light of our analysis, we proposed an experimental strategy to test the existence of birhythmicity in the p53-Mdm2 network. This strategy relies on a key characteristic of multistable systems which is to convert a transient stimulus into a permanent response of the cell. However, one may wonder what the physiological interest of birhythmicity in the p53 response to DNA damage is. One possible advantage could be to enhance the response of the system in contrast to mono-rhythmic systems which may require large perturbations and long time to induce a significant frequency change. Birhythmicity would thus allow a better flexibility of the p53 response by permitting the cell to quickly switch from one oscillatory regime to another after DNA damage.

## Materials and Methods

The simulations are performed with XPPAUT (http://www.math.pitt.edu/~bard/xpp/xpp.html) for Model 1 and with Matlab for Model 2 and 3.

## Supporting Information

Table S1 **Equations of evolution for Model 2.** The domains D^ij^ of the phase space are delimited by the threshold values of the step functions: K_P_, K_Mn_ and K_Mc_ (Figure 5A).
(DOC)

Table S2 **Target equilibrium points for Model 2.** In each domain D^ij^, the equations of evolution are linear and the Jacobian matrix is triangular with negative elements in the diagonal (see Table S1). In each domain D^ij^ of the phase space, the system will thus tend towards the target equilibrium of D^ij^.
(DOC)

Table S3 **Equations of evolution for Model 3.** The domains D^ij^ of the phase space are delimited by the threshold values of the step functions K_P_, K_Mn_, K_Mc_ and the additional threshold K. Mc has been set as a constant Mc^ij^ in each domain D^ij^.
(DOC)

Table S4 **Conditions on the parameter values of Model 3 to respect constraint (1).** The focal points have been chosen such that the transition graph contains the two embedded cycles composing the graph of transitions of Model 2 (Figure 6): one cycle crossing respectively domains D^14^, D^24^, D^23^ and D^13^; one cycle crossing respectively domains D^14^, D^24^, D^23^, D^22^, D^21^, D^11^, D^12^ and D^13^ (Figure 7). The conditions on the parameter values of Model 3 to respect constraint (1) can then be directly derived.
(DOC)

Text S1 **The OAK Model.**
(DOC)

Text S2 **Estimation of the terms modeling the translocation of nuclear Mdm2 to the cytoplasm and the Mdm2-mediated acceleration of p53 degradation in the OAK Model.**
(DOC)

Text S3 **Analytical study of Model 1.**
(DOC)

Text S4 **Comparison between the bifurcation diagrams of Model 1 and Model 2.**
(DOC)

Text S5 **Conditions on the parameter values of Model 3 to respect constraint (2).**
(DOC)

Text S6 **Analytical expression of the first return map for Model 3.**
(DOC)

Figure S1 **Bifurcation diagram and projection of the phase portrait of birhythmicity in the plane (Mn,P) for the OAK model.** (A) Bifurcation diagrams of p53 level as a function of d_Mn_ for the OAK Model (Ouattara et al., 2010). Solid lines (resp. dashed lines) represent the stable (resp. unstable) equilibrium points. Bold (resp. white) dots are the maxima and minima of the stable (resp. unstable) limit cycles. The system shows a birhythmic domain for 1.09 h^-1^<d_Mn_<1.3h^−1^. (B) Projection of the two oscillatory regimes on the plane (Mn,P) for d_Mn_ = 1.2 h^−1^. The thresholds, K_Mc_ and K_Mn_, related to P are indicated in dashed lines. The parameter values for the bifurcation diagram are K_Mn_ = 0.1 nM, K_Mc_ = 0.6 nM, k_P_ = 5 nM.h^−1^, k_Mc_ = 0.1 nM.h^−1^, k'_Mc_ = 1.2 nM.h^−1^, d_P_ = 0.1 h^−1^, d'_P_ = 2.3 nM^−1^.h^−1^, d_Mc_ = 0.6 h^−1^, k_in_ = 0.45 h^−1^, k'_in_ = 0.4 h^−1^, k_out_ = 0.045 h^−1^, K_P_ = 0.2 nM and Vr = 10.
(DOC)

Figure S2
**Study of the number of equilibrium points for Model 1.** Graph of the rational function R (left) and a zoom of this graph (right) for the parameter values indicated in Figure 3. The roots of R give the equilibrium points for Model 1. The equilibrium points are included in the interval [0; 
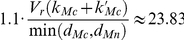
].(DOC)
