# Supplementary material for: A Theoretical Exploration of Birhythmicity in the p53-Mdm2 Network
Source: PLoS One. 2011 Feb 14;6(2):e17075. doi: 10.1371/journal.pone.0017075 (PMC3038873; doi:10.1371/journal.pone.0017075)
Supplement: Table S1 — Equations of evolution for Model 2. The domains Dij of the phase space are delimited by the threshold values of the step functions: KP, KMn and KMc (Figure 5A). (DOC) [file pone.0017075.s001.doc]

| **Domain D^13^**  **P>K_Mc_ and Mn<K_P_** | **Domain D^23^**  **P>K_Mc_ and Mn>K_P_** |
| --- | --- |
| **Domain D^12^**  **K_Mn_<P<K_Mc_ and Mn<K_P_** | **Domain D^22^**  **K_Mn_<P<K_Mc_ and Mn>K_P_** |
| **Domain D^11^**  **P<K_Mn_ and Mn<K_P_** $\begin{matrix} \frac{\text{d}P}{\text{d}t}=k_{P}-d_{P}\cdot P \\ \frac{\text{d}Mc}{\text{d}t}=k_{Mc}-\left(k_{in}+d_{Mc}\right)\cdot Mc \\ \frac{\text{d}Mn}{\text{d}t}=V_{r}\cdot k_{in}\cdot Mc-d_{Mn}Mn \end{matrix}$ | **Domain D^21^**  **P<K_Mn_ and Mn>K_P_** $\begin{matrix} \frac{\text{d}P}{\text{d}t}=-d_{P}\cdot P \\ \frac{\text{d}Mc}{\text{d}t}=k_{Mc}-\left(k_{in}+d_{Mc}\right)\cdot Mc \\ \frac{\text{d}Mn}{\text{d}t}=V_{r}\cdot k_{in}\cdot Mc-d_{Mn}Mn \end{matrix}$ |
