# Supplementary material for: A Theoretical Exploration of Birhythmicity in the p53-Mdm2 Network
Source: PLoS One. 2011 Feb 14;6(2):e17075. doi: 10.1371/journal.pone.0017075 (PMC3038873; doi:10.1371/journal.pone.0017075)
Supplement: Table S2 — Target equilibrium points for Model 2. In each domain Dij, the equations of evolution are linear and the Jacobian matrix is triangular with negative elements in the diagonal (see Table S1). In each domain Dij of the phase space, the system will thus tend towards the target equilibrium of Dij. (DOC) [file pone.0017075.s002.doc]

| **Domain** | **Target equilibrium point** |
| --- | --- |
| **D^11^** | $\begin{matrix} P^{11}=\frac{k_{P}}{d_{P}} \\ Mc^{11}=\frac{k_{Mc}}{k_{in}+d_{Mc}} \\ Mn^{11}=\frac{V_{r}\cdot k_{in}}{d_{Mn}}\cdot\frac{k_{Mc}}{k_{in}+d_{Mc}} \end{matrix}$ |
| **D^21^** | $\begin{matrix} P^{21}=0 \\ Mc^{21}=\frac{k_{Mc}}{k_{in}+d_{Mc}} \\ Mn^{21}=\frac{V_{r}\cdot k_{in}}{d_{Mn}}\cdot\frac{k_{Mc}}{k_{in}+d_{Mc}} \end{matrix}$ |
| **D^12^** |  |
| **D^22^** |  |
| **D^13^** |  |
| **D^23^** |  |
