# Supplementary material for: A Theoretical Exploration of Birhythmicity in the p53-Mdm2 Network
Source: PLoS One. 2011 Feb 14;6(2):e17075. doi: 10.1371/journal.pone.0017075 (PMC3038873; doi:10.1371/journal.pone.0017075)
Supplement: Table S3 — Equations of evolution for Model 3. The domains Dij of the phase space are delimited by the threshold values of the step functions KP, KMn, KMc and the additional threshold K. Mc has been set as a constant Mcij in each domain Dij. (DOC) [file pone.0017075.s003.doc]

| **Domain D^14^**  **P>K_Mc_ and Mn<K_P_** | **Domain D^24^**  **P>K_Mc_ and Mn>K_P_** |
| --- | --- |
| **Domain D^13^**  **K_Mn_<P<K_Mc_ and Mn<K_P_** | **Domain D^23^**  **K_Mn_<P<K_Mc_ and Mn>K_P_** |
| **Domain D^12^**  **K<P<K_Mn_ and Mn<K_P_** $\begin{matrix} \frac{\text{d}P}{\text{d}t}=k_{P}-d_{P}\cdot P \\ \frac{\text{d}Mn}{\text{d}t}=V_{r}\cdot k_{in}\cdot Mc^{_{12}}-d_{Mn}Mn \end{matrix}$ | **Domain D^22^**  **K<P<K_Mn_ and Mn>K_P_** $\begin{matrix} \frac{\text{d}P}{\text{d}t}=-d_{P}\cdot P \\ \frac{\text{d}Mn}{\text{d}t}=V_{r}\cdot k_{in}\cdot Mc^{_{22}}-d_{Mn}Mn \end{matrix}$ |
| **Domain D^11^**  **P<K and Mn<K_P_** $\begin{matrix} \frac{\text{d}P}{\text{d}t}=k_{P}-d_{P}\cdot P \\ \frac{\text{d}Mn}{\text{d}t}=V_{r}\cdot k_{in}\cdot Mc^{_{11}}-d_{Mn}Mn \end{matrix}$ | **Domain D^21^**  **P<K and Mn>K_P_** $\begin{matrix} \frac{\text{d}P}{\text{d}t}=-d_{P}\cdot P \\ \frac{\text{d}Mn}{\text{d}t}=V_{r}\cdot k_{in}\cdot Mc^{_{21}}-d_{Mn}Mn \end{matrix}$ |
