# Supplementary material for: A Theoretical Exploration of Birhythmicity in the p53-Mdm2 Network
Source: PLoS One. 2011 Feb 14;6(2):e17075. doi: 10.1371/journal.pone.0017075 (PMC3038873; doi:10.1371/journal.pone.0017075)
Supplement: Table S4 — Conditions on the parameter values of Model 3 to respect constraint (1). The focal points have been chosen such that the transition graph contains the two embedded cycles composing the graph of transitions of Model 2 (Figure 6): one cycle crossing respectively domains D14, D24, D23 and D13; one cycle crossing respectively domains D14, D24, D23, D22, D21, D11, D12 and D13 (Figure 7). The conditions on the parameter values of Model 3 to respect constraint (1) can then be directly derived. (DOC) [file pone.0017075.s004.doc]

| **Domain** | **Focal point** | **Conditions on the focal points to respect constraint (1)** |
| --- | --- | --- |
| **D^11^** | $\begin{matrix} P^{11}=\frac{k_{P}}{d_{P}} \\ Mn^{11}=\frac{V_{r}\cdot k_{in}\cdot Mc^{11}}{d_{Mn}} \end{matrix}$ | $\begin{matrix} P^{11}\geq K \\ Mn^{11}\leq K_{P} \end{matrix}$ |
| **D^21^** | $\begin{matrix} P^{21}=0 \\ Mn^{21}=\frac{V_{r}\cdot k_{in}\cdot Mc^{21}}{d_{Mn}} \end{matrix}$ | $\begin{matrix} P^{21}\leq K \\ Mn^{21}\leq K_{P} \end{matrix}$ |
| **D^12^** | $\begin{matrix} P^{12}=\frac{k_{P}}{d_{P}} \\ Mn^{12}=\frac{V_{r}\cdot k_{in}\cdot Mc^{12}}{d_{Mn}} \end{matrix}$ | $\begin{matrix} P^{12}\geq K_{Mn} \\ Mn^{12}\leq K_{P} \end{matrix}$ |
| **D^22^** | $\begin{matrix} P^{22}=0 \\ Mn^{22}=\frac{V_{r}\cdot k_{in}\cdot Mc^{22}}{d_{Mn}} \end{matrix}$ | $\begin{matrix} P^{22}\leq K \\ Mn^{22}\geq K_{P} \end{matrix}$ |
| **D^13^** |  | $\begin{matrix} P^{13}\geq K_{Mc} \\ Mn^{13}\leq K_{P} \end{matrix}$ |
| **D^23^** |  | $\begin{matrix} P^{23}\leq K_{Mn} \\ Mn^{23}\leq K_{P} \end{matrix}$ |
| **D^14^** |  | $\begin{matrix} P^{14}\geq K_{Mc} \\ Mn^{14}\geq K_{P} \end{matrix}$ |
| **D^24^** |  | $\begin{matrix} P^{24}\leq K_{Mc} \\ Mn^{24}\geq K_{P} \end{matrix}$ |
