# Supplementary material for: A Theoretical Exploration of Birhythmicity in the p53-Mdm2 Network
Source: PLoS One. 2011 Feb 14;6(2):e17075. doi: 10.1371/journal.pone.0017075 (PMC3038873; doi:10.1371/journal.pone.0017075)
Supplement: Text S1 — The OAK Model. (DOC) [file pone.0017075.s005.doc]

We recall the differential equations which describe the model of the p53-Mdm2 core network of Ouattara et al. [1] (OAK Model):

where P, Mc and Mn represent the concentration of p53, cytoplasmic Mdm2 and nuclear Mdm2 respectively.

The terms $d_{P}\cdot P$and $d_{P}'\cdot Mn\cdot P$ represent the basal and Mdm2-mediated degradation of p53, respectively. $k_{P}\frac{K_{P}^{n}}{K_{P}^{n}+Mn^{n}}$ refers to the production of p53. The term $k_{Mc}$ corresponds to the basal production of Mdm2 whereas $k'_{Mc}\frac{P^{n}}{K_{Mc}^{n}+P^{n}}$ relates to the p53-dependent production of cytoplasmic Mdm2. $d_{Mc}Mc$ represents the degradation of cytoplasmic Mdm2. The terms $\frac{1}{V_{r}}k_{out}\cdot Mn$ and $k_{out}\cdot Mn$ model the translocation process of nuclear Mdm2 into the cytoplasm whereas refers to the nuclear import of cytoplasmic Mdm2 (inhibited by p53). $d_{Mn}Mn$ corresponds to the degradation of nuclear Mdm2, which is accelerated by DNA damage. Finally, Vr represents the ratio between the cytoplasmic and the nuclear volume and appears in the translocation processes involved in the model. As p53 forms a tetramer to enhance the expression of its target genes [2-4], Hill functions with exponent n=4 have been used to model the processes involving p53 activity in the OAK Model.

It should be noted that the incorporation of the nonlinear term $k_{P}\frac{K_{P}^{n}}{K_{P}^{n}+Mn^{n}}$ which models the downregulation of the production of p53 may not be fully justified on biological grounds.

However, removing this term and using a nonlinear, Goldbeter–Koshland function [5] for the Mdm2-mediated degradation of p53 leads to a bifurcation picture as a function of d_Mn_ which is very similar to the bifurcation diagram of the OAK Model (see Figure A1 in [1]). Moreover, in the domain of birhythmicity, the shape of the orbits of the two limit cycles is also very similar to the one for the OAK Model (not shown).

**References**

1.Ouattara DA, Abou-Jaoudé W, Kaufman M (2010) From structure to dynamics: frequency tuning in the p53-Mdm2 network. II Differential and stochastic approaches. J Theor Biol 264: 1177-1189.

2.Chène P (2001) The role of tetramerization in p53 function. Oncogene 20:2611-2617.

3.McLure KG, Lee PW (1998) How p53 binds DNA as a tetramer. EMBO J 17:3342-3350.

4.Weinberg RL, Veprintsev DB, Fersht AR (2004) Cooperative binding of tetrameric p53 to DNA. J Mol Biol 341:1145-1159.

5.Goldbeter A, Koshland DE (1981) An amplified sensitivity arising from covalent modification in biological systems. Proc Natl Acad Sci USA 78:6840-6844.
