# Supplementary material for: A Theoretical Exploration of Birhythmicity in the p53-Mdm2 Network
Source: PLoS One. 2011 Feb 14;6(2):e17075. doi: 10.1371/journal.pone.0017075 (PMC3038873; doi:10.1371/journal.pone.0017075)
Supplement: Text S2 — Estimation of the terms modeling the translocation of nuclear Mdm2 to the cytoplasm and the Mdm2-mediated acceleration of p53 degradation in the OAK Model. (DOC) [file pone.0017075.s006.doc]

a) We compared the term $k_{out}\cdot Mn$ with the other terms of the equation of evolution of Mn for the parameter values indicated in Figure S1. In the oscillatory domain (Figure S1, left), $d_{Mn}\geq0.5h^{-1}$ and$k_{out}=0.045h^{-1}$. The term $k_{out}\cdot Mn$ is thus small compared to the term $d_{Mn}\cdot Mn$ of the equation of Mn ($k_{out}\leq0.1\cdot d_{Mn}$) in the domain where the system displays oscillations. $k_{out}\cdot Mn$ is thus predicted to have little effect on the dynamics of our system in the oscillatory domain.

b) We also compared the term $\frac{1}{V_{r}}k_{out}\cdot Mn$ with the other terms of the equation of Mc along the orbits of the limit cycles for the parameter values indicated in Figure S1. In the oscillatory domain (Figure S1, left), we can delimit a domain in the phase space, containing the orbit of the oscillatory regimes, in which Mn<2nM (not shown). In this domain, $\frac{1}{V_{r}}k_{out}\cdot Mn$~0.01 nM.h^-1^. This term is small compared to the basal production of Mdm2, k_Mc_ (k_Mc_=0.1 nM.h^-1^) suggesting that the term $\frac{1}{V_{r}}k_{out}\cdot Mn$ has little effect on the oscillatory dynamics of the system.

c) The inhibition of p53 by nuclear Mdm2 is modeled by two terms in the OAK Model, one of them (the Hill term $k_{P}\frac{K_{P}^{4}}{K_{P}^{4}+Mn^{4}}$) being much more nonlinear than the other (the bilinear term $d_{P}'\cdot Mn\cdot P$) (see Text S1). Since the emergence of complex behaviors such as oscillations and birhythmicity is closely related to high enough nonlinearities, we predict that the bilinear term has little influence on the oscillatory dynamics of the system compared with the Hill term.
