# Supplementary material for: A Theoretical Exploration of Birhythmicity in the p53-Mdm2 Network
Source: PLoS One. 2011 Feb 14;6(2):e17075. doi: 10.1371/journal.pone.0017075 (PMC3038873; doi:10.1371/journal.pone.0017075)
Supplement: Text S3 — Analytical study of Model 1. (DOC) [file pone.0017075.s007.doc]

*1-Model 1 is equivalent to a competitive system*

The sign of the elements of the Jacobian matrix of Model 1 is:

$$\left(\begin{matrix} - & 0 & - \\ + & - & 0 \\ - & + & - \end{matrix}\right)$$

After the change of variable, Mc to -Mc, the signs of the elements of the Jacobian matrix become:

$$\left(\begin{matrix} - & 0 & - \\ - & - & 0 \\ - & - & - \end{matrix}\right)$$

which is a competitive matrix because the off-diagonal terms of the Jacobian matrix are non positive.

*2-Study of the number of equilibrium points for Model 1*

The equations of Model 1 at steady state are the following:

(1) $0=k_{P}\frac{K_{P}^{4}}{K_{P}^{4}+Mn_{ss}^{4}}-d_{P}\cdot P_{ss}$

(2)

(3)

where P_SS_, Mc_SS_ and Mn_SS_ represent the steady state concentration of P, Mc and Mn respectively.

The equilibrium points of Model 1 are thus the solutions of the above set of equations. We next eliminate P_ss_ and Mc_ss_ variables in order to get an equation which depends solely on Mn_ss_.

From (1), we can express P_ss_ as a function of Mn_ss_

(4)$P_{ss}=k_{P}\frac{K_{P}^{4}}{d_{P}\cdot\left(K_{P}^{4}+Mn_{ss}^{4}\right)}=\Psi\left(Mn_{ss}\right)$

From (3) and (4), we can express Mc_ss_ as a function of Mn_ss_

(5)

By replacing Mc_ss_ and P_ss_ in (2) by their expression in (4) and (5) as functions of Mn_ss_, we get:(6)

After simplification, we finally obtain:

(7)with $\Psi\left(Mn_{ss}\right)=k_{P}\frac{K_{P}^{4}}{d_{P}\cdot\left(K_{P}^{4}+Mn_{ss}^{4}\right)}$

R(0) is positive,$\lim_{x\to+\infty} R\left(x\right)=-\infty$and R is continuous in R^3^_+_. Therefore, R has at least one root in R^3^_+_ so at least one equilibrium point in R^3^_+_.

We next searched bounds for Mn_ss_ value. By multiplying the equation of evolution of cytoplasmic Mdm2 by Vr and adding it to the equation of nuclear Mdm2, we obtain:

Thus, we can fix ε_0_ as small as we want such that we have for t sufficiently high:

By taking for example ε_0_=0.1, we obtain that the steady state of Mn, Mn_ss_, belongs to the interval: I=[0, ].

The graph of R, for the parameter values indicated in Figure 3, is presented in Figure S2. The graph shows only one zero in the interval I. Therefore, for these parameter values, Model 1 presents a unique equilibrium point.

As seen above, Model 1 is equivalent to a competitive system. Therefore, the system has one real negative eigenvalue in R^3^_+_ [1]. A numerical analysis further shows that, at the equilibrium point and for the parameter values indicated in Figure 3, the two other eigenvalues are complex with a positive real part (not shown). Therefore, the equilibrium point is unstable and presents a 1-dimensional stable manifold.

**Reference**

1.Hirsch MW (1988) Systems of differential equations which are competitive or cooperative: III. Competing species. Nonlinearity 1:51-71.
