# Supplementary material for: A Theoretical Exploration of Birhythmicity in the p53-Mdm2 Network
Source: PLoS One. 2011 Feb 14;6(2):e17075. doi: 10.1371/journal.pone.0017075 (PMC3038873; doi:10.1371/journal.pone.0017075)
Supplement: Text S4 — Comparison between the bifurcation diagrams of Model 1 and Model 2. (DOC) [file pone.0017075.s008.doc]

We first derive the conditions on the parameter values for Model 2 to have a stable equilibrium point in domain D^21^ (low p53 level, high nuclear Mdm2 level) and the conditions to have a stable equilibrium point in domain D^13^ (high p53 level, low nuclear Mdm2 level).

To have a stable equilibrium point in domain D^21^, the target equilibrium point of D^21^ (Table S2) has to belong to D^21^. This leads to the following conditions:

$\frac{V_{r}\cdot k_{in}}{d_{Mn}}\cdot\frac{k_{Mc}}{k_{in}+d_{Mc}}\geq K_{P}$ which leads to $d_{Mn}\leq\frac{V_{r}\cdot k_{in}}{K_{P}}\cdot\frac{k_{Mc}}{k_{in}+d_{Mc}}=d_{1}$

Similarly, to have a stable equilibrium point in domain D^13^, the target equilibrium point of D^13^ (Table S2) has to belong to D^13^. This leads to the following conditions:

$\frac{k_{P}}{d_{P}}\geq K_{Mc}$ and

which leads to :

$\frac{k_{P}}{d_{P}}\geq K_{Mc}$ and

For the parameter values indicated in Figure 3, the condition $\frac{k_{P}}{d_{P}}\geq K_{Mc}$is fulfilled and d_1_<d_2_ (d_1_~2.1 h^-1^ and d_2_=5 h^-1^). Therefore, for low d_Mn_ values (d_Mn_<d_1_), the system presents a stable equilibrium point of low level of p53. For high d_Mn_ values (d_Mn_>d_2_), the system presents a stable equilibrium point of high level of p53. For intermediate d_Mn_ values (d_1_<d_Mn_<d_2_), numerical simulations shows that the system presents an oscillatory regime with a domain of birhythmicity (Figure 5). The bifurcation picture of Model 2 as a function of d_Mn_ is thus consistent with the bifurcation diagram of Model 1 (Figure 3A).
