# Supplementary material for: A Theoretical Exploration of Birhythmicity in the p53-Mdm2 Network
Source: PLoS One. 2011 Feb 14;6(2):e17075. doi: 10.1371/journal.pone.0017075 (PMC3038873; doi:10.1371/journal.pone.0017075)
Supplement: Text S5 — Conditions on the parameter values of Model 3 to respect constraint (2). (DOC) [file pone.0017075.s009.doc]

To respect constraint (2), Mc^ij^ values have been chosen such as:

- Mc^11^<Mc^21^<Mc^22^<Mc^23^<Mc^24^ and Mc^24^>Mc^14^>Mc^13^>Mc^12^>Mc^11^. For these conditions, the parameter values Mc^ij^ are in accordance with the evolution of Mc in the large amplitude limit cycle in Model 2 (Figure 8C and 8D);
- Mc^13^<Mc^14^<Mc^24^ and Mc^24^>Mc^23^>Mc^13^. For these conditions, the parameter values Mc^ij^ are in accordance with the evolution of Mc in the small amplitude limit cycle in Model 2 (Figure 8A and 8B).
