# Supplementary material for: A Theoretical Exploration of Birhythmicity in the p53-Mdm2 Network
Source: PLoS One. 2011 Feb 14;6(2):e17075. doi: 10.1371/journal.pone.0017075 (PMC3038873; doi:10.1371/journal.pone.0017075)
Supplement: Text S6 — Analytical expression of the first return map for Model 3. (DOC) [file pone.0017075.s010.doc]

1. *Derivation of the analytical expression of the first return map in the [0, x) axis for Model 3*

To compute the first return map F(x) from and to the [0, x) axis (Figure 10), we consider the point (K_P_, K_Mc_+x_0_) with x_0_>0 as an initial condition and compute the function F(x_0_) such that (K_P_, K_Mc_+F(x_0_)) is the image of (K_P_, K_Mc_+x_0_) on the [0, x) axis after one cycle in the phase space.

Since the degradation constants d_Mn_ and d_P_ have the same values, the trajectories in each domain are straight lines containing the focal point of the domain. If the trajectory enters domain D^ij^ from a point (x_0_,y_0_) and if (a^ij^,b^ij^) is the focal point of D^ij^, then the equations of the trajectory in D^ij^ is:

$y=\left(\frac{y_{0}-b^{ij}}{x_{0}-a^{ij}}\right)\cdot x+\left(\frac{b^{ij}x_{0}-a^{ij}y_{0}}{x_{0}-a^{ij}}\right)$ (1)

As the transition graph contains two embedded cycles which both cross the [0, x) axis (Figure 7), we have to compute the first return map for each of the two cycles. To derive the conditions on x_0_ to follow either the small amplitude or the large amplitude cycle of the transition graph, we first have to compute the image of a point of the [0, x) axis, (K_P_, x), in the half-line of equation {(x,y)/x>K_P_, y=K_Mc_} (i.e. boundary between domains D^23^ and D^24^) (point (f_1_(x), K_Mc_)), and the image of the point (f_1_(x), K_Mc_), in the segment of equation {(x,y)/x=K_P_, K_Mn_<y<K_Mc_}(i.e. boundary between domains D^13^ and D^23^) (point (K_P_, f_2_(f_1_(x)))). From equation (1), we obtain the analytical expression of f_1_ and f_2_:

$f_{1}\left(x\right)=\frac{\text{a}^{24}x+\text{K}_{\text{Mc}}\text{K}_{\text{P}}\text{-b}^{24}\text{K}_{\text{P}}}{x\text{-b}^{24}\text{+K}_{\text{Mc}}}$ and $f_{2}\left(x\right)=\frac{\text{-b}^{23}x+\text{K}_{\text{P}}\text{b}^{23}\text{-K}_{\text{P}}\text{K}_{\text{Mc}}\text{+a}^{23}\text{K}_{\text{Mc}}}{\text{-}x+\text{a}^{23}}$

which are homographic functions.

If f_2_(x)>K_Mn_, the trajectory will firstly cross the boundary separating D^13^ and D^23^ and will follow the small amplitude cycle in the transition graph. Otherwise, the trajectory will firstly cross the boundary separating D^22^ and D^23^ and will follow the large amplitude cycle in the transition graph. We can thus derive the conditions on x_0_ to follow either the small or the large amplitude cycle:

$f_{2}\left(f_{1}\left(x_{0}\right)\right)>K_{Mn}$ to follow the small amplitude cycle

$f_{2}\left(f_{1}\left(x_{0}\right)\right)<K_{Mn}$ to follow the large amplitude cycle

Since $f_{2}^{\circ}f_{1}$ is a continuous and strictly decreasing function, we can define its inverse function $\left(f_{2}^{\circ}f_{1}\right)^{-1}$. This yields to the following conditions:

$0<x_{0}<x_{D}$ to follow the small amplitude cycle,

$x_{0}>x_{D}$ to follow the large amplitude cycle,

with . For the parameter values indicated in Figure 9, we have x_D_~0.077.

For the small amplitude cycle, we can define the image of:

- a point (K_P_, x), (f_3_(x), K_Mc_), for K_Mn_<x<K_Mc_ and 0<f_3_(x)< K_P_
- a point (x, K_Mc_), (K_P_, f_4_(x)), in the [0, x) axis for 0<x<K_P_.

Similarly, for the large amplitude cycle, we can define the image of:

- a point (x, K_Mc_), (f_5_(x), K_Mn_), for x>K_P_ and f_5_(x)>K_P_
- a point (x, K_Mn_), (f_6_(x), K), for x>K_P_ and f_6_(x)>K_P_
- a point (x, K), (K_P_, f_7_(x)), for x>K_P_ and 0<f_7_(x)<K
- a point (K_P_, x), (f_8_(x), K), for 0<x<K and f_8_(x)<K_P_
- a point (x, K), (f_9_(x), K_Mn_), for 0<x<K_P_ and 0<f_9_(x)< K_P_
- a point (x, K_Mn_), (f_10_(x), K_Mc_), for 0<x<K_P_ and 0<f_10_(x)<K_P_

From equation (1), we obtain the analytical expression of f_3_, f_4_, f_5_, f_6_, f_7_, f_8_, f_9_, f_10_ and f_11_:

$$f_{3}\left(x\right)=\frac{\text{a}^{13}x-\text{K}_{\text{Mc}}\text{a}^{13}+\text{K}_{\text{Mc}}\text{K}_{\text{p}}\text{-b}^{13}\text{K}_{\text{p}}}{x-\text{b}^{13}}$$

$$f_{4}\left(x\right)=\frac{\text{b}^{14}x\text{-K}_{\text{P}}\text{b}^{14}\text{+K}_{\text{P}}\text{K}_{\text{Mc}}\text{-a}^{14}\text{K}_{\text{Mc}}}{x\text{-a}^{14}}$$

$$f_{5}\left(x\right)=\frac{\left(\text{K}_{\text{Mn}}\text{-b}^{23}\right)\cdot x\text{-K}_{\text{Mn}}\text{a}^{23}\text{+a}^{23}K_{\text{Mc}}}{K_{\text{Mc}}\text{-b}^{23}}$$

$$f_{6}\left(x\right)=\frac{\left(\text{K-b}^{22}\right)\cdot x\text{-Ka}^{22}\text{+a}^{22}\text{K}_{\text{Mn}}}{\text{K}_{\text{Mn}}\text{-b}^{22}}$$

$$f_{7}\left(x\right)=\frac{\text{-b}^{21}x+\text{K}_{\text{P}}\text{b}{}^{21}\text{-K}_{\text{P}}\text{K+a}^{21}\text{K}}{\text{a}^{21}\text{-}x}$$

$$f_{8}\left(x\right)=\frac{\text{-a}^{11}x+\text{Ka}^{11}\text{-KK}_{\text{P}}\text{+b}^{11}\text{K}_{\text{P}}}{x\text{-b}^{11}}$$

$$f_{9}\left(x\right)=\frac{\left(\text{K}_{\text{Mn}}\text{-b}^{12}\right)\cdot x\text{-K}_{\text{Mn}}\text{a}^{12}\text{+a}^{12}\text{K}}{\text{K-b}^{12}}$$

$$f_{10}\left(x\right)=\frac{\left(\text{K}_{\text{Mc}}\text{-b}^{13}\right)\cdot x\text{-K}_{\text{Mc}}\text{a}^{13}\text{+a}^{13}\text{K}_{\text{Mn}}}{\text{K}_{\text{Mn}}\text{-b}^{13}}$$

Therefore, the analytical expression of the first return map, F(x), from and to the [0, x) axis is:

$F\left(x\right)=f_{4}\left(f_{3}\left(f_{2}\left(f_{1}\left(x\right)\right)\right)\right)$ for $0<x<x_{D}$

$F\left(x\right)=f_{4}\left(f_{10}\left(f_{9}\left(f_{8}\left(f_{7}\left(f_{6}\left(f_{5}\left(f_{1}\left(x\right)\right)\right)\right)\right)\right)\right)\right)$ for $x>x_{D}$

F is thus a composition of homographic functions. It is thus also a homographic function in each of the two intervals of definition. Indeed, if we compose two homographic functions, $f\left(x\right)=\frac{ax+b}{cx+d}$ and $g\left(x\right)=\frac{a'x+b'}{c'x+d'}$, we have:

$$\begin{matrix} f\left(g\left(x\right)\right)=\frac{a\frac{a'x+b'}{c'x+d'}+b}{c\frac{a'x+b'}{c'x+d'}+d}=\frac{\frac{a\left(a'x+b'\right)+b\left(c'x+d'\right)}{c'x+d'}}{\frac{c\left(a'x+b'\right)+d\left(c'x+d'\right)}{c'x+d'}} \\ \begin{matrix} \begin{matrix} & \end{matrix} & \end{matrix}=\frac{a\left(a'x+b'\right)+b\left(c'x+d'\right)}{c\left(a'x+b'\right)+d\left(c'x+d'\right)}=\frac{\left(aa'+bc'\right)x+\left(ab'+bd'\right)}{\left(ca'+dc'\right)x+\left(cb'+dd'\right)} \end{matrix}$$

which is also homographic.

For the parameter values indicated in Figure 9, we obtain:

$F\left(x\right)\approx\frac{x}{\text{0.23+16}\cdot x}$ for $0<x<0.077$ (2)

$F\left(x\right)\approx\frac{0.1+0.3\cdot x}{1.1+2.9\cdot x}$ for $x>0.077$ (3)

According to the definition of F, the fixed points of F (i.e. points x for which F(x)=x) correspond to periodic orbits of the system crossing the [0, x) axis. From equations (2) and (3), we prove that F admits two strictly positive fixed points, x_1_ and x_2_ (see Figure 10), with x_1_~0.048 and x_2_~0.093.

Calculating the left-handed and the right-handed limit of F when x tends to x_D_, we get:

$\lim_{x\to x_{D}^{-}} \left(F\left(x\right)\right)\approx0.054$ and $\lim_{x\to x_{D}^{+}} \left(F\left(x\right)\right)\approx0.094$

Therefore, F admits a point of discontinuity at x=x_D_.

1. *Derivation of the analytical expression of the first return map in the [0, x) axis for the modified model 3*

To compute the first return function F’(x) from and to the [0, x) axis for the modified Model 3 (Figure 11), we also have to define the image of:

- a point (x, K_Mn_), (K_P_, f_11_(x)), for x>K_P_ and K<f_11_(x)<K_Mn_,
- a point (K_P_, x), (f_12_(x), K_Mn_), for K<x<K_Mn_ and 0<f_12_(x)<K_P_.

in addition to the functions defined above for the calculation of the first return map, F, of Model 3.

From equation (1), we obtain:

$$f_{11}\left(x\right)=\frac{\text{b}^{22}x\text{-K}_{\text{P}}\text{b}^{22}\text{+K}_{\text{P}}\text{K}_{\text{Mn}}\text{-a}^{22}\text{K}_{\text{Mn}}}{x\text{-a}^{22}}$$

$$f_{12}\left(x\right)=\frac{\text{a}^{12}x\text{-K}_{\text{Mn}}\text{a}^{12}+\text{K}_{\text{Mn}}\text{K}_{\text{P}}\text{-b}^{12}\text{K}_{\text{P}}}{x-\text{b}^{12}}$$

As the transition graph contains now three embedded cycles each crossing the [0, x) axis (Figure 11A), we have to compute the first return map for each of the three cycles. The conditions on x_0_ to follow either the small, the intermediate or the large amplitude cycle are:

$f_{2}\left(f_{1}\left(x_{0}\right)\right)>K_{Mn}$ to follow the small amplitude cycle

$f_{2}\left(f_{1}\left(x_{0}\right)\right)<K_{Mn}$ and $f_{11}\left(f_{5}\left(f_{1}\left(x_{0}\right)\right)\right)>K$to follow the intermediate amplitude cycle

$f_{11}\left(f_{5}\left(f_{1}\left(x_{0}\right)\right)\right)<K$to follow the large amplitude cycle

This yields to the following conditions:

$0<x_{0}<x_{D}$ to follow the small amplitude cycle,

$x_{D}<x_{0}<x_{E}$ to follow the intermediate amplitude cycle,

$x_{E}<x_{0}$ to follow the large amplitude cycle,

with

The analytical expression of the first return map, F’(x), from and to the [0, x) axis is:

$F'\left(x\right)=f_{4}\left(f_{3}\left(f_{2}\left(f_{1}\left(x\right)\right)\right)\right)$ for $0<x<x_{D}$

$F'\left(x\right)=f_{4}\left(f_{10}\left(f_{12}\left(f_{11}\left(f_{5}\left(f_{1}\left(x\right)\right)\right)\right)\right)\right)$ for $x_{D}<x<x_{E}$

$F'\left(x\right)=f_{4}\left(f_{10}\left(f_{9}\left(f_{8}\left(f_{7}\left(f_{6}\left(f_{5}\left(f_{1}\left(x\right)\right)\right)\right)\right)\right)\right)\right)$ for $x_{E}<x$

For the parameter values indicated in Figure 11, we prove that F’ admits only one strictly positive fixed point, x_1_, (see Figure 11) with x_1_~0.048 (results not shown).

The left-handed and the right-handed limit of F’ when x tends to x_D_ is the same (not shown). Therefore, F’ is continuous at x=x_D_. However, calculating the left-handed and the right-handed limit of the derivative of F’ when x tends to x_D_ for the parameter values indicated in Figure 11, we obtain:

$\lim_{x\to x_{D}^{-}} \frac{dF}{dx}\left(x\right)\approx0.108$ and $\lim_{x\to x_{D}^{+}} \frac{dF}{dx}\left(x\right)\approx0.105$

The two limits are different. Therefore, F’ admits a non smooth point at x=x_D_.
