# Supplementary figures and images for: A Theoretical Exploration of Birhythmicity in the p53-Mdm2 Network
Source: PLoS One. 2011 Feb 14;6(2):e17075. doi: 10.1371/journal.pone.0017075 (PMC3038873; doi:10.1371/journal.pone.0017075)

| **A**  **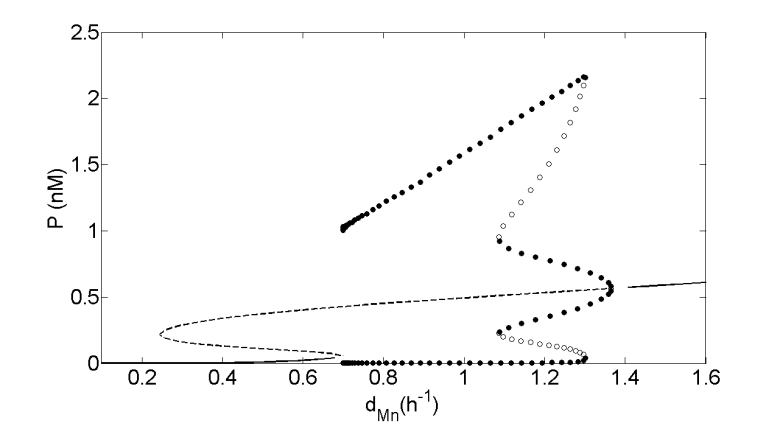** | **B**  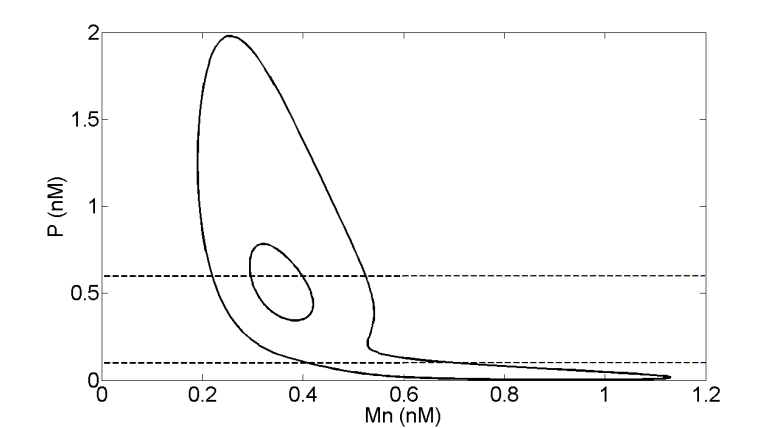 |
| --- | --- |

Supplement: Figure S1 — Bifurcation diagram and projection of the phase portrait of birhythmicity in the plane (Mn,P) for the OAK model. (A) Bifurcation diagrams of p53 level as a function of dMn for the OAK Model (Ouattara et al., 2010). Solid lines (resp. dashed lines) represent the stable (resp. unstable) equilibrium points. Bold (resp. white) dots are the maxima and minima of the stable (resp. unstable) limit cycles. The system shows a birhythmic domain for 1.09 h-1<dMn<1.3h−1. (B) Projection of the two oscillatory regimes on the plane (Mn,P) for dMn = 1.2 h−1. The thresholds, KMc and KMn, related to P are indicated in dashed lines. The parameter values for the bifurcation diagram are KMn = 0.1 nM, KMc = 0.6 nM, kP = 5 nM.h−1, kMc = 0.1 nM.h−1, k'Mc = 1.2 nM.h−1, dP = 0.1 h−1, d'P = 2.3 nM−1.h−1, dMc = 0.6 h−1, kin = 0.45 h−1, k'in = 0.4 h−1, kout = 0.045 h−1, KP = 0.2 nM and Vr = 10. (DOC) [file pone.0017075.s011.doc]

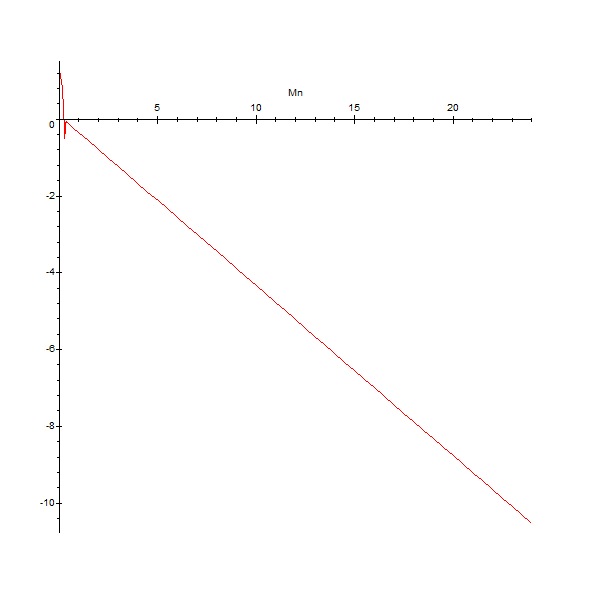

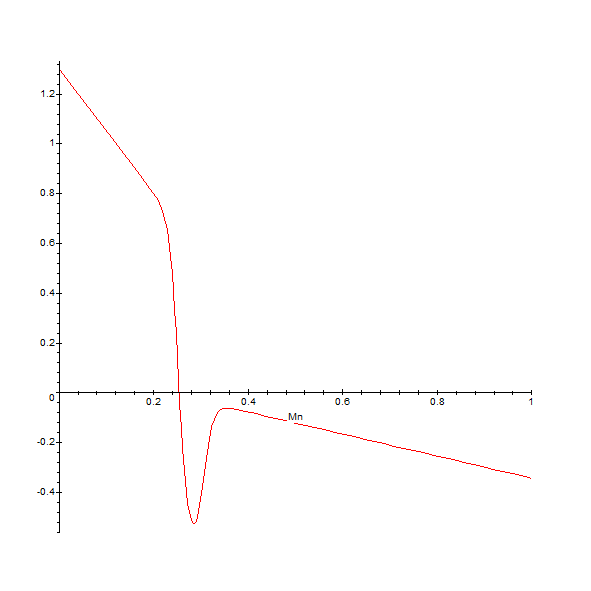

Supplement: Figure S2 — Study of the number of equilibrium points for Model 1. Graph of the rational function R (left) and a zoom of this graph (right) for the parameter values indicated in Figure 3. The roots of R give the equilibrium points for Model 1. The equilibrium points are included in the interval [0; ]. (DOC) [file pone.0017075.s012.doc]
